# Simultaneous Ipsilateral Vascularized Lymph Node Transplantation and Contralateral Lymphovenous Anastomosis in Bilateral Extremity Lymphedema with Different Severities

**DOI:** 10.1245/s10434-020-08720-2

**Published:** 2020-06-18

**Authors:** M.-H. Cheng, R. Tee, C. Chen, C.-Y. Lin, M. Pappalardo

**Affiliations:** 1 Department of Plastic and Reconstructive Surgery, Chang Gung Memorial Hospital, College of Medicine, Chang Gung University, Taoyuan, Taiwan; 2 Center for Tissue Engineering, Chang Gung Memorial Hospital, Taoyuan, Taiwan; 3 Department of General Surgery, Cedars Sinai Medical Center, Los Angeles, CA USA; 4 Plastic and Reconstructive Surgery, Department of Surgical, Oncological and Oral Sciences, University of Palermo, Palermo, Italy

## Abstract

**Background:**

Extremity lymphedema can occur bilaterally with different severities on each side. The aim of this study is to investigate the treatment outcomes of such patients with bilateral extremity lymphedema of different severities.

**Patients and Methods:**

Between 2013 and 2017, patients with bilateral extremity lymphedema of different severities according to the Taiwan Lymphoscintigraphy Staging (TLS) system were retrospectively reviewed. Ipsilateral vascularized lymph node transplantation (VLNT) was indicated in TLS total obstruction and contralateral lymphovenous anastomosis (LVA) in TLS partial obstruction with patent lymphatic vessels on indocyanine green lymphography. Outcomes were assessed using circumference improvement, frequency of cellulitis, and lymphedema-specific quality of life (LYMQoL) questionnaires.

**Results:**

A total of 10 patients with bilateral extremity lymphedema with median age of 63 (range 12–75) years were included. The median symptom duration of the lymphedematous limb was 60 (range 36–168) months and 12 (range 1–60) months in the VLNT and LVA group, respectively (*p* < 0.05). At average follow-up of 37.5 (range 14–58) months, the average limb circumference improvement was 2.4 (range − 3.3 to 7.8) cm in the VLNT group and 2.3 (range 0.3–7) cm in the LVA group (*p* = 1). The median episodes of cellulitis decreased significantly from 4 to 0.5 and 1 to 0 times/year in the VLNT and LVA group, respectively (*p *= 0.02, *p* = 0.06). The overall LYMQoL score improved from 4.5 preoperatively to 7.5 postoperatively (*p* < 0.01).

**Conclusions:**

Limb-specific VLNT and LVA selected by TLS effectively treated bilateral extremity lymphedema with different severities.

Secondary extremity lymphedema is a burdensome sequela experienced by cancer survivors.^1^ Lymphedema management typically consists of complex decongestive therapy, which is only partially effective and does not prevent the progression of extremity lymphedema.^2^ Advances in lymphedema microsurgery including lymphovenous anastomosis (LVA)^3^ and vascularized lymph node transplantation (VLNT)^4^ have yielded promising outcomes over the last decade. Both techniques redirect stagnant lymph in the lymphedematous limb to the venous system, which is achieved by directly shunting the lymph into a subdermal venule in the LVA technique or by bypassing lymph through transplanted lymph nodes in VLNT.^5^^,^^6^

Independently, the selection of LVA versus VLNT for treatment of extremity lymphedema has been the subject of academic debate across centers owing to a variance in disease assessments and plans, including the presentations of severity, staging/grading systems, insurance limitations, patient preferences, and even surgeon experience.^6^ The general consensus of experts on lymphedema microsurgery is that LVA is more effective in the earlier stages of lymphedema while VLNT is commonly indicated in more advanced cases.^7^ A more complex scenario is when extremity lymphedema develops simultaneously in both limbs but with different stages of severity in each, representing a unique challenge for procedure selection. The first treatment of primary bilateral extremity lymphedema by LVA in one limb and VLNT in the other limb for different severities of lymphedema was reported in 2014.^8^ An objective tool to evaluate the severity of extremity lymphedema and select an appropriate surgical treatment is Cheng’s Lymphedema Grading (CLG), which utilizes clinical circumferential measurement to assess the normal extremity as a control in unilateral extremity lymphedema.^9^^,^^10^ Consequently, as understanding of the pathophysiology of extremity lymphedema deepens, the evaluations and treatments of different lymphedemas have been extended and the applicability of current assessment tools also needs to be explored. However, there is a lack of reports and guidelines for evaluating and selecting the appropriate surgical treatment for patients with bilateral extremity lymphedema. This condition is inherently an important form of clinical lymphedema that can benefit from investigative guidelines, as it is inappropriate to assume that patients with differing limb lymphedema severities can be adequately treated with a single surgical procedure.

The aim of this study is to investigate the patient selection and treatment outcomes of ipsilateral VLNT and contralateral LVA for individual lymphedematous limb in patients with bilateral extremity lymphedema of different severities.

## Patients and Methods

This single-institutional retrospective study of patients with bilateral extremity lymphedema who underwent ipsilateral VLNT and contralateral LVA between November 2013 and October 2018 was approved by the Chang Gung Memorial Hospital Institutional Review Board (IRB: 201800600B0). Lymphoscintigraphy and indocyanine green (ICG) lymphography were parts of the standard preoperative work-up and assisted in evaluating extremity lymphedema severity and in guiding the selection of the appropriate surgical procedures. Magnetic resonance imaging (MRI) was used to evaluate the donor-site lymph node basins. Doppler ultrasound was used to assess the recipient-site vessels.

Patients with bilateral extremity lymphedema of different severities in both limbs according to the Taiwan Lymphoscintigraphy Staging (TLS) system were indicated to undergo ipsilateral VLNT (VLNT group) and contralateral LVA (LVA group).^8^^,^^9^^,^^11^ Lymphedematous limbs with longer symptom duration (> 5 years) and that were stage P-3 or total obstruction stages T-4, T-5, or T-6 according to the Taiwan Lymphoscintigraphy Staging system but did not have patent lymphatic ducts on ICG lymphography underwent vascularized submental lymph node (VSLN) flap transfer.^8^^,^^9^^,^^11^^,^^12^ Lymphedematous limbs with relatively short-term symptoms (< 5 years), partial obstruction in Taiwan Lymphoscintigraphy stages P-1, P-2, or P-3, and patent lymphatic ducts identified on ICG lymphography were selected for LVA.^13^^,^^14^ Patients with unilateral extremity lymphedema and those with bilateral lymphedema who underwent the same procedures bilaterally for both limbs were excluded.^8^

The prospectively collected data included patient demographics, lymphedema etiology, duration of symptoms prior to treatment, compliance with complex decongestive therapy, TLS severity of both limbs, and episodes of cellulitis. Compliance with complex decongestive therapy was defined as use of grade 2 compression garments for at least 12 h per day in addition to daily manual lymphatic drainage. Patient limb circumferential measurements were taken preoperatively and postoperatively at every clinical visit and were measured at 10 cm above and below the elbow joint for the upper limbs and 15 cm proximal and distal to the patella for the lower limbs. While the circumferential measurements were utilized in surgical outcome comparisons, they were not incorporated into circumferential differences or circumferential reduction rate, since there was no “healthy” limb to serve as a baseline.^10^

Changes in the circumference of each extremity from before to after the operation were documented. Preoperative versus postoperative comparisons were also carried out for the number of episodes of cellulitis per year.

### Lymphedema-Specific Quality of Life (LYMQoL) Questionnaire

Upper or lower extremity lymphedema-specific quality of life (LYMQoL) questionnaires were administered both preoperatively and at 12 months postoperatively to patients receiving upper or lower limb treatment, respectively.^9^–^11^^,^^15^^,^^16^ The questionnaires consisted of 27 questions (upper extremity) or 28 questions (lower extremity) covering four domains (function, appearance, symptoms, and mood). A lower score in each domain corresponds to better QoL in that aspect. The overall patient QoL was scored on a scale of 0 to 10, with 10 being the best QoL score (inverse to the individualized domains of LYMQoL).

### Surgical Techniques

A VSLN flap was harvested by a modified technique with partial preservation of the medial platysma, as described previously.^17^^,^^18^ The dorsal wrist and ankle were the preferred recipient sites of the upper and lower extremity transfers, respectively, as described by Cheng et al.^4^^,^^17^ Key maneuvers included careful microscopic dissection of the branches of the marginal mandibular nerve, sparing of the medial platysma, 5 cm in width, to avoid marginal mandibular nerve pseudoparalysis,^18^ vigilance for regional vascular anatomical variations, and optimization in harvesting the maximal number of submental lymph nodes available in the region.^19^

The side-to-end LVA technique performed by the senior author (M.-H.C.) was previously described with preoperative ICG lymphography evaluation of the patent lymphatic vessels available.^13^^,^^14^ Use of ultrasound for the evaluation of the lymphatic vessels and recipient venules was not available at our hospital.^20^^,^^21^ One or two side-to-end anastomoses between a lymphatic channel and subdermal venule were executed preferentially under 42× magnification using a Mitaka MM50 microscope (Kohki Co, Ltd., Tokyo, Japan) for early-grade lymphedematous limbs. Anastomoses were performed using 11-0 Nylon sutures (Ethicon, New Brunswick, NJ). ICG lymphography was used to map the lymphatic ducts preoperatively and intraoperatively to confirm anastomosis patency.^13^^,^^22^

### Postoperative Care

After simultaneously receiving VLNT and LVA, patients were asked to comply with a standardized 2-week rehabilitation program upon discharge, consisting of progressive muscle strength training and reverse manual proximal-to-distal lymphatic drainage. Patients did not wear any compression garments or bandages postoperatively on either limb.

### Statistics

The data are presented as median and range for continuous variables. The statistical analyses were performed using SPSS 21.0 statistical software (SPSS, Inc., Chicago, Ill.). The nonparametric Mann–Whitney *U* test was used for comparisons between the VLNT and LVA groups. Preoperative and postoperative differences were analyzed by Wilcoxon matched-pairs signed-rank tests. A *p* value ≤ 0.05 was considered statistically significant.

## Results

A total of ten patients with bilateral extremity lymphedema with different severities between the lymphedematous limbs who underwent ipsilateral VLNT and contralateral LVA at the same time were included. All ten patients were female, with median age of 63 (range 12–75) years preoperatively (Table 1). Three patients had primary bilateral lower extremity lymphedema. Five patients with bilateral lower extremity lymphedema received adjuvant radiation therapy to the pelvis but presented with lower extremity lymphedema of different severities. Two bilateral breast cancer patients received adjuvant radiation therapy after axillary lymph node dissection in unilateral extremity, which was subjected to a VLNT procedure. In the LVA group, partial obstruction of TLS P-1 was present in four limbs, while P-2 was observed in six limbs. In the VLNT group, total obstruction of TLS T-4 was present in four limbs, and T-5 was observed in six limbs. The average symptom duration and preoperative duration of CDT were 60 (range 36–168) months and 12 (range 0–24) months in the VLNT group, and 12 (range 1–60) months and 4.8 (range 0–24) months in the LVA group (*p* < 0.05 and < 0.05, respectively) (Table 2).

**Table 1:** Demographics and etiology of ten patients with bilateral extremity lymphedema

	Age (years)	Preop. BMI (kg/m^2^)	Etiology (change to radiation, chemotherapy)
Case 1	75	27.4	Cervical cancer with hysterectomy and RT
Case 2	64	30.0	Endometrial cancer with hysterectomy and PLND + RT + CT
Case 3	71	30.6	Endometrial cancer with hysterectomy and BSO + LND + RT + CT
Case 4	60	19.9	Right breast cancer with mastectomy and ALND + RT
			Left breast cancer with mastectomy and CT
Case 5	61	23.1	Right breast cancer with mastectomy and ALND + CT + RT
			Left breast cancer with mastectomy and ALND + CT
Case 6	63	25.4	Endometrial cancer with hysterectomy + BSO + PLND + RT + CT
Case 7	63	24.2	Primary lymphedema
Case 8	63	22.1	Cervical cancer with hysterectomy and RT
Case 9	60	26.3	Primary lymphedema
Case 10	12	26.8	Primary lymphedema
Median (range)	63 (12–75)	25.9 (19.9–30.6)	

*RT* radiotherapy, *ALND* axillary lymph node dissection, *CT* chemotherapy, *BSO* bilateral salpingo-oophorectomy, *PLND* pelvic lymph node dissection

**Table 2:** Characteristics of bilateral extremity lymphedema subjected to ipsilateral vascularized lymph node transplantations and contralateral lymphovenous anastomoses

Case no. (limb)	Limbs affected	Side	Procedure	Duration		Lymphoscintigraphy		ICG
				Symptoms (months)	CDT (months)	Pattern	Stage	Patent lymphatic ducts (yes/no)
1	Lower	L	LVA	12	0	Partial obstruction	P-1	Yes
2	Lower	R	LVA	60	24	Partial obstruction	P-2	Yes
3	Lower	R	LVA	36	0	Partial obstruction	P-1	Yes
4	Upper	L	LVA	1	0	Partial obstruction	P-2	Yes
5	Upper	L	LVA	1	0	Partial obstruction	P-2	Yes
6	Lower	R	LVA	14	12	Partial obstruction	P-2	Yes
7	Lower	L	LVA	6	0	Partial obstruction	P-1	Yes
8	Lower	L	LVA	3	0	Partial obstruction	P-1	Yes
9	Lower	R	LVA	3	0	Partial obstruction	P-2	Yes
10	Lower	R	LVA	120	36	Partial obstruction	P-2	Yes
Subtotal of LVA, median (range)				12* (1–60)	4.8* (0–24)			
1	Lower	R	VLNT	168	6	Total obstruction	T-5	No
2	Lower	L	VLNT	60	24	Total obstruction	T-4	No
3	Lower	L	VLNT	36	0	Total obstruction	T-5	No
4	Upper	R	VLNT	36	12	Total obstruction	T-5	No
5	Upper	R	VLNT	84	24	Total obstruction	T-5	No
6	Lower	L	VLNT	36	24	Total obstruction	T-4	No
7	Lower	R	VLNT	36	12	Total obstruction	T-4	No
8	Lower	R	VLNT	12	6	Total obstruction	T-5	No
9	Lower	L	VLNT	240	240	Total obstruction	T-5	No
10	Lower	L	VLNT	120	36	Total obstruction	T-4	No
Subtotal of VLNT, median (range)				60* (36–168)	12* (0–24)			

*LVA* lymphovenous anastomosis, *CDT* complex decongestive therapy, *VLNT* vascularized lymph node transplantation

*Statistically significant, *p* < 0.05

### Response to Lymphedema Microsurgeries

Ten limbs underwent a successful VSLN flap, to the ankle in eight limbs and the wrist in two limbs, with a 100% flap success rate. One of the VSLN flaps required reexploration due to venous congestion, which was successfully salvaged with a vein reanastomosis (case 3). The average preoperative body mass index (BMI) of 25.9 (range 19.9–30.6) kg/m^2^ was statistically improved to 23.7 kg/m^2^ (range 19.7–29.9) kg/m^2^ postoperatively (*p* = 0.01). All patients who received a VSLN flap did not develop facial lymphedema or marginal mandibular nerve palsy. One side-to-end anastomosis was performed in nine extremities and two anastomoses were performed in one extremity in the LVA group, giving a mean of 1.1 anastomoses. There were no complications associated with any of the LVAs.

One patient (case 7) developed a pelvic recurrence 10 months postoperatively. At an average follow-up time of 37.5 (range 14–58) months, the average limb circumference decreased by 2.4 (range − 3.3 to 7.8) cm and 2.3 (range 0.3–7) cm in the VLNT and LVA group, respectively (*p* = 1) (Table 3). Six of the eight (75%) limbs in the VLNT group and eight of eight (100%) in the LVA group showed circumferential improvement after operations, respectively (Table 3), excluding one case with recurrence and one case younger than 12 years old. Case 3 who had an unsatisfactory outcome in the limb underwent VLNT due to venous compromise, but not in the LVA limb (Table 3). The average number of episodes of cellulitis decreased significantly from 4 (range 0–5) to 0.5 (range 0–3) times/year (*p *= 0.02) in the VLNT group, and from 1 (range 0–2) to 0 (range 0–1) times/year (*p *= 0.06) in the LVA group.

**Table 3:** Outcomes of treatment post ipsilateral vascularized lymph node transplantations and contralateral lymphovenous anastomoses

Case no.	BMI (kg/m^2^)		Frequency of cellulitis (times per year)				Circumference improvement (cm)						Follow-up
			VLNT		LVA		VLNT			LVA			
	Preop.	Postop.	Preop.	Postop.	Preop.	Postop.	AE/AK	BE/BK	Average	AE/AK	BE/BK	Average	Months
1	27.4	24.9	5	1	0	0	5	10.5	7.8	4	0.5	2.3	45
2	30.0	28.7	0	0	2	0	4	3	3.5	3	1.5	2.3	39
3	30.6	29.9	5	3	2	1	– 3	– 3.5	– 3.3	2	0.5	1.3	37
4	19.9	19.7	5	0	0	0	3.5	– 0.5	1.5	1.5	1	0.3	38
5	23.1	23.2	2	0	2	0	2.5	4.0	3.3	2	4	3	21
6	25.4	22.1	2	1	2	1	1	0	0.5	3	0.5	1.8	58
7^a^	24.2	22.2	4	1	0	0	3.5	– 5	– 0.8	2	–3	0.5	16
8	22.1	23.1	4	0	0	0	0	– 2.5	– 1.3	5	0.5	2.8	22
9	26.3	24.2	4	0	0	0	14	0	7	12	2	7	14
10^b^	26.8	26.2	5	1	2	0	– 2	– 1.5	– 1.8	4.5	– 1	1.8	45
Median (range)	25.9 (19.9–30.6)	23.7 (19.7–29.9)	4 (0–5)	0.5 (0–3)	1 (0–2)	0 (0–1)	3 (– 3 to 14)	0 (– 3.5 to 10.5)	2.4 (– 3.3 to 7.8)	3 (1.5–12)	0.5 (1–4)	2.3 (0.3–7)	37.5 (14–58)
*p* value	0.01*		0.02*		0.06		1						

*AK* above knee, *AE* above elbow, *BK* below knee, *BE* below elbow, *AA* above ankle

*Statistically significant, *p* < 0.05

^a^This patient developed pelvic recurrence at 10 months postoperatively, being excluded from the total/median calculation since it influenced the bilateral lower limb lymphedema

^b^This 12-year-old patient was excluded from the total/median calculation since she was still in natural growth in height and weight, which influenced the assessment of circumferences of bilateral lower extremities

### Improvement in LYMQoL

**Fig. 1:**
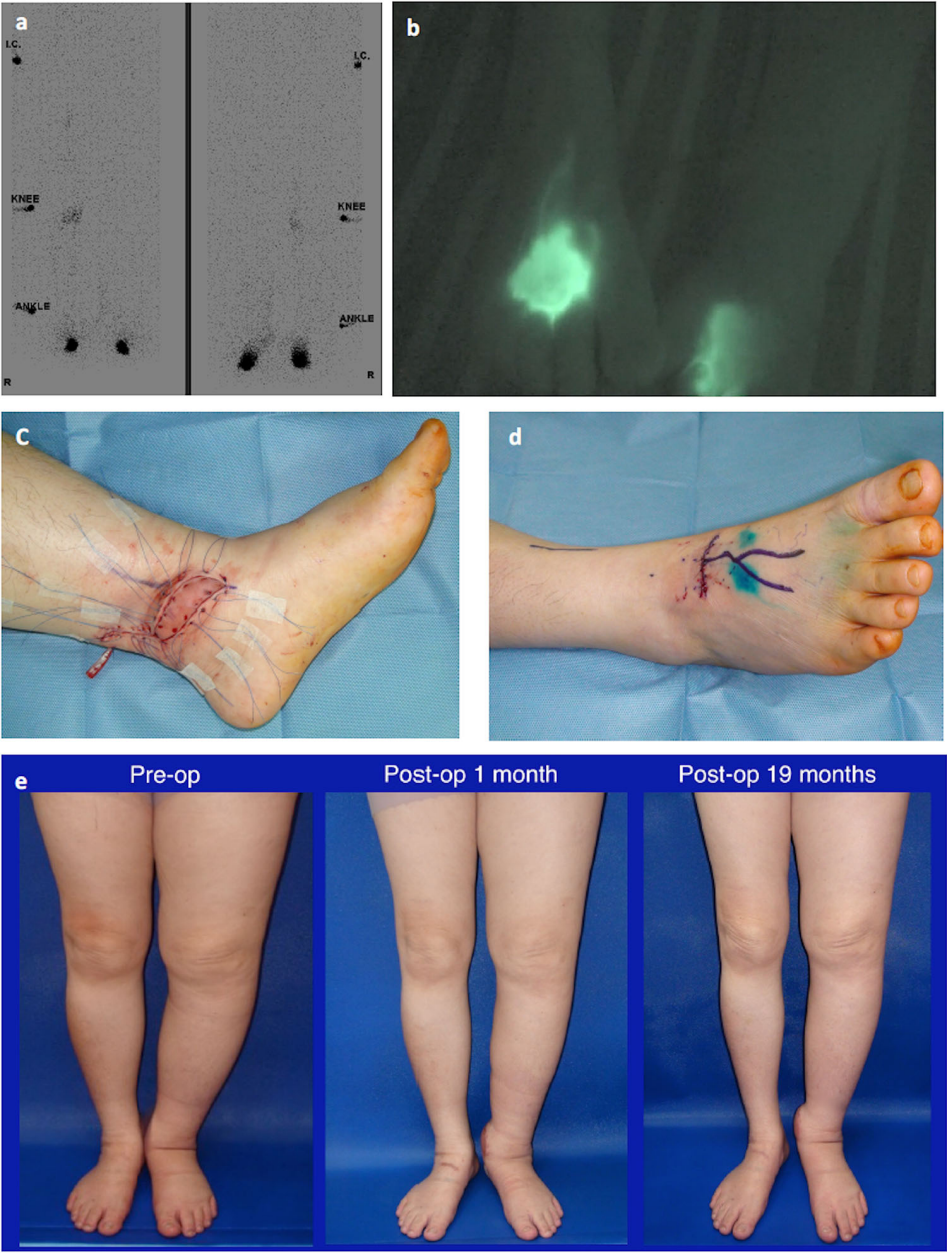
Diagnosis, surgical treatment, and outcome of a 68-year-old female (case 2) who suffered from bilateral lower extremity lymphedema of different severities. **a** Lymphoscintigraphy showed bilateral lymphedema, and the Taiwan Lymphoscintigraphy Staging results were more severe on the left (total obstruction) than right side (partial obstruction); **b** Indocyanine green lymphography showed patent lymphatic ducts in the right leg but diffuse dermal backflow in the left leg; **c** A right vascularized submental lymph node flap was harvested and transferred to left ankle with end-to-end anastomosis of the facial artery to the posterior tibial artery and end-to-end anastomosis of the facial vein to the great saphenous vein; **d** Two side-to-end lymphovenous anastomoses were performed on the right ankle; **e** Photographs taken preoperatively and at 19-month follow-up showed improvement in the right leg (at 4 and 3 cm above and below knee) and left leg (at 3 and 1.5 cm above and below knee). Her overall LYMQoL improved from 5 to 8 at 12-month follow-up

The preoperative LYMQoL scores showed high levels of functional impairment and morbidity with median scores of 34 (range 30–38) in function, 28 (range 19–28) in appearance, 20 (range 18–24) in symptoms, and 24 (range 22–24) in mood. At mean follow-up of 12 months, the scores of all domains improved, with 18 (range 16–25) in function (*p* < 0.01), 10 (range 8–20) in appearance (*p* < 0.01), 10 (range 8–18) in symptoms (*p* < 0.01), and 10 (range 7–18) in mood (*p* < 0.01). The overall LYMQoL score showed improvement from a score of 4.5 (range 2–5) preoperatively to 7.5 (range 6–8) postoperatively (*p* < 0.01) (Fig. 1) (Table 4).

**Table 4:** Lymphedema-specific quality of life (LYMQOL) measurement of ten patients who underwent ipsilateral vascularized lymph node transplantations and contralateral lymphovenous anastomoses

Case no.	Function (ULL: 10–40) (LLL: 8–32)		Appearance (ULL: 5–20) (LLL: 7–28)		Symptoms (ULL: 10–40) (LLL: 5–20)		Mood (ULL: 10–40) (LLL: 6–24)		Overall quality of life (0–10)	
	Preop.	Postop. 12 months	Preop.	Postop. 12 months	Preop.	Postop. 12 months	Preop.	Postop. 12 months	Preop.	Postop. 12 months
1	32	20	28	12	20	10	24	10	2	7
2	30	16	27	10	20	8	23	7	5	8
3	31	25	28	20	20	17	24	12	5	6
4	37	18	19	9	22	10	24	9	5	8
5	38	24	20	15	24	18	24	18	4	8
6	32	20	28	10	22	10	22	10	3	6
7	37	16	28	12	22	8	22	9	5	7
8	36	16	25	10	23	10	23	10	4	6
9	37	16	28	8	24	8	24	10	5	8
10	30	18	28	10	24	11	24	10	5	8
Median (range)	34 (30–38)	18 (16–25)	28 (19–28)	10 (8–20)	20 (18–24)	10 (8–18)	24 (22–24)	10 (7–18)	4.5 (2–5)	7.5 (6–8)
Preop. versus postop. 12 months *p* value	< 0.01*		< 0.01*		< 0.01*		< 0.01*		< 0.01*	

*LYMQOL* lymphedema-specific quality of life measurement

*Statistically significant, *p* < 0.05

## Discussion

Ten patients with bilateral extremity lymphedema were evaluated using the TLS staging system and ICG lymphography, found to have asymmetric severity in the individual limb, and given limb-individualized treatment involving ipsilateral VLNT and contralateral LVA simultaneously. To the best of the authors’ knowledge, this study is the first case series of patients with bilateral extremity lymphedema of different severities who underwent VLNT in the advanced-stage lymphedematous extremity and LVA in the early-stage extremity. The results of this study reveal that the aforementioned procedures individualized by disease severity achieved efficacious improvement in limb circumference and episodes of cellulitis for each limb, as well as improvements in overall LYMQoL.

Methodologically, the evaluation modalities were critical for understanding and standardizing the degree of severity in bilateral lymphedematous extremities in the absence of a “normal” baseline. Lymphoscintigraphy yields useful information about proximal and intermediate lymph nodes, superficial and deep lymphatic ducts, and dermal backflow.^1^^,^^9^^,^^23^ ICG lymphography, specific the Dermal Backflow ICG Classification by Yamamoto et al., is a crucial additional component in the evaluation modalities, as it reveals the patency of superficial lymphatic ducts and dermal backflow patterns.^3^^,^^24^ We found that using the linear pattern of ICG as the indication for LVA in the extremity lymphedema with partial obstruction on lymphoscintigraphy enabled great functional recovery of the early-stage extremity lymphedema. Real-time flow dynamics and visualization of the course of lymphatics offer data that is complementary and confirmatory to the results of ICG lymphography in addition to the generalized defects visualized by lymphoscintigraphy. By utilizing both lymphoscintigraphy and ICG lymphography to classify the lymphedema severity in individual limb, the indications of VLNT and LVA for individual lymphedematous limb could be clarified and executed effectively.^7^^,^^25^^,^^26^

Interestingly, five patients with lower extremity lymphedema included in this study showed different progression of lymphedema between the two lower limbs after the same pelvic interventions (Table 1).^1^^,^^27^^,^^28^ This observation suggests that it is possible that these patients developed partial obstruction in unilateral lower extremity lymphedema but total obstruction in the other lower extremity.^24^

In case 3, in which circumferential improvement after the VSLN flap transfer was not found, venous congestion with successful salvage was encountered. This patient had multiple comorbidities including history of deep venous thrombosis and chronic diabetes, which are predisposing conditions for venous thrombosis and postoperative infection. Such conditions can compromise functional recovery after VLNT.

In the LVA group, anastomoses were performed in side-to-end fashion to avoid damaging existing functional lymphatic ducts. A mean of 1.1 LVAs per limb was performed in this study to achieve success in the early-stage lymphedematous limbs without performing VLNT.^13^^,^^14^ Interestingly, Tourani et al. showed that the long-term patency rates of LVA in chronic lymphedema animal models are suboptimal.^29^ This lack of durability is hypothesized to be due to chronic inflammation and scarring of the lymphatics. This issue is not a known problem with distal VLNT for advanced-stage lymphedematous limbs, where transferred vascularized lymph nodes actively drain the lymph into the venous system by the pump mechanism, catchment effect, and gravity effects.^5^^,^^6^

Although durable results are typically a standard goal for surgical interventions, it may not be appropriate to suggest that only VLNT be performed for bilateral extremity lymphedema regardless of the stage of severity. First, VLNT has been shown to be indicated for advanced-stage extremity lymphedema, and it is a more invasive procedure than LVA. Compared with LVA, VLNT requires intraoperative techniques of greater complexity and is associated with a higher risk for postoperative reexploration due to the nature of the multiple anastomoses and the need to maintain lymph node viability and the flap inset.^30^ Additionally, the submental region is the preferred donor lymph node basin,^11^^,^^17^ as it includes numerous sizable lymph nodes, a reliable skin paddle, and minimal risk for iatrogenic lymphedema.^9^^,^^11^^,^^31^^,^^32^ Bilateral LVAs for both lymphedematous limbs are potentially alternative treatments for bilateral extremity lymphedema, as this does not require a second procedure (removal of the skin paddle in VLNT). This is unlikely to be an adequate solution as LVA has been shown to be less efficacious than VLNT in higher stages of lymphedema.^11^ Additionally, in more severe or late-stage limbs, the level of scarring, fibrosis, and inflammation in the affected limb can be severe and increase the risk that the performed LVA will damage the residual lymphatic vessels and thus of an unnecessary and ineffective procedure. Thus, it is more rational to tailor the treatment of both lymphedematous limbs on an individual basis by lymphatic severity, even in the context of bilateral extremity lymphedema with a common cause.

Proper resolution of extremity lymphedema in individual limb is of the utmost importance. Extremity lymphedema has been shown to compromise the quality of life of patients and hinder their activities. As the survival rate of cancer patients continues to improve, it will become increasingly important to strive to improve the quality of life of cancer survivors; thus, it is imperative to improve lymphedema treatment.^10^ All patients in this study experienced significant overall quality of life improvement.

The major limitation of this study is its small sample size, partly reflective of limiting selection criteria. Longer follow-up times and a large number of patients are mandatory in further studies.

## Conclusions

Lymphoscintigraphy and ICG lymphography can accurately differentiate the different severity in the individual limb of bilateral extremity lymphedema patients. Simultaneous ipsilateral VLNT and contralateral LVA were effective for bilateral extremity lymphedema with asymmetrical severity, resulting in improvements in circumferential measurements, episodes of cellulitis, and LYMQoL.

## Data Availability

The data that support the findings of this study are available from the corresponding author upon reasonable request.
